# Evidence for a pervasive ‘idling-mode’ activity template in flying and pedestrian insects

**DOI:** 10.1098/rsos.150085

**Published:** 2015-05-20

**Authors:** Andrew M. Reynolds, Hayley B. C. Jones, Jane K. Hill, Aislinn J. Pearson, Kenneth Wilson, Stephan Wolf, Ka S. Lim, Don R. Reynolds, Jason W. Chapman

**Affiliations:** 1 Rothamsted Research, Harpenden, Hertfordshire AL5 2JQ, UK; 2 Department of Biology, University of York, York YO10 5DD, UK; 3 Lancaster Environment Centre, Lancaster University, Lancaster LA1 4YQ, UK; 4 School of Biological and Chemical Sciences, Queen Mary University of London, London E1 4NS, UK; 5 Natural Resources Institute, University of Greenwich, Chatham, Kent ME4 4TB, UK; 6 Environment and Sustainability Institute, University of Exeter, Penryn, Cornwall TR10 9EZ, UK

**Keywords:** spontaneous movement patterns, intermittent locomotion, behavioural bursts, Lévy flights, power-law distributions

## Abstract

Understanding the complex movement patterns of animals in natural environments is a key objective of ‘movement ecology’. Complexity results from behavioural responses to external stimuli but can also arise spontaneously in their absence. Drawing on theoretical arguments about decision-making circuitry, we predict that the spontaneous patterns will be scale-free and universal, being independent of taxon and mode of locomotion. To test this hypothesis, we examined the activity patterns of the European honeybee, and multiple species of noctuid moth, tethered to flight mills and exposed to minimal external cues. We also reanalysed pre-existing data for *Drosophila* flies walking in featureless environments. Across these species, we found evidence of common scale-invariant properties in their movement patterns; pause and movement durations were typically power law distributed over a range of scales and characterized by exponents close to 3/2. Our analyses are suggestive of the presence of a pervasive scale-invariant template for locomotion which, when acted on by environmental cues, produces the movements with characteristic scales observed in nature. Our results indicate that scale-finite complexity as embodied, for instance, in correlated random walk models, may be the result of environmental cues overriding innate behaviour, and that scale-free movements may be intrinsic and not limited to ‘blind’ foragers as previously thought.

## 2. Introduction

Several recent research studies have reported universal statistical laws which characterize how resting and active periods are interwoven throughout human daily life [1–7]. Before these studies it was widely believed that human activities were randomly distributed in time and were thus well approximated by Poisson processes, so that consecutive events tend to follow each other at relatively regular time intervals, and very long periods were essentially precluded. It is now recognized that active and resting period durations do, in fact, follow universal distributions with ‘heavy’ power-law tails that lack characteristic scales [1–7]. These statistical laws have important practical applications; for example, the inherent similarities underpinning human travel patterns could impact all phenomena driven by human mobility, from epidemic prevention to emergency response, urban planning and agent-based modelling [5], while similarities in waiting behaviours have important implications from resource management to service allocation in both communications and retail [1]. The occurrence of these statistical laws is remarkable given that human behaviours are strongly shaped by an individual's psyche and by complex social and environmental interactions. The presence of these activity patterns holds promise that similar universal laws may also describe non-human animal movements. The identification of such laws, and the mechanisms that underpin them, is a key objective of movement ecology [8]. Complex animal movement patterns result from behavioural responses to external stimuli, but they can also arise spontaneously when external stimuli are limited (hereafter called ‘null movement patterns’). Such null movements are likely to be universal because they are intrinsically cued, and understanding them will help to elucidate how complex movement patterns can arise in nature. Studies of null patterns do not ignore environmental influences, but rather seek to establish whether and how complex movement patterns can arise in their absence.

Drawing on theoretical arguments about decision-making circuitry, we predict that the spontaneous patterns will be scale-free and universal, being independent of taxon and mode of locomotion. We therefore hypothesize that intermittent scale-free movement patterns are innate and realized when external cues that elicit behavioural responses are minimal, and that scale-finite behaviours are triggered by external cues. This is congruent with the Lévy flight hypothesis, which posits that foragers should have scale-free movement patterns when searching in the absence of environmental cues [9,10]. To test this hypothesis we analysed new activity data from a wide range of insect species: (i) 15 species of wild-caught common British noctuid moths (Lepidoptera), (ii) laboratory-reared *Spodoptera exempta* (African armyworm) noctuid moths, and (iii) *Apis mellifera* (European honeybee) workers (Hymenoptera), all flown on flight mills under conditions of limited external cues. We also re-analyse previously published data for the walking movement patterns of *Drosophila* *melanogaster* flies (Diptera) within circular homogeneous arenas [11]. The diversity of these taxa (18 species from three insect orders) and locomotion modes (flying and walking) allows us to test decisively for the presence of a universal null movement pattern in the absence of external stimuli. Our analyses use an array of statistical tests that together can reliably distinguish between power laws and strongly competing alternative models of our movement pattern [12–15]. Power-law scaling typically extends over two or more decades. This fulfils Stumpf & Porter's [16] ‘rule of thumb’; after critically appraising power laws identified in biological systems, they suggested that a candidate power-law probability frequency distribution should apply over at least 2 orders of magnitude along both axes. In the case of cumulative frequency distributions used herein this equates to at least one order of magnitude along the ordinate, and at least two orders of magnitude along the abscissa.

Previous studies [17–20] hint at the presence of a universal statistical law for null movements in non-humans that is closely analogous to that found to describe human activities [1,5,6]. The case was, however, not compelling owing to various limitations in the previous studies. Among the first to examine animal motion under limited external cues were Cole [17] and Martin [18], who reported on null movement patterns in individual adult *D. melanogaster* fruit flies moving within featureless, unchanging arenas. These studies [17,18] reported that the ‘null movements’ were interspersed with pauses and that the pause durations had scale-free distributions with heavy power-law tails—a trait also seen in some rodent species in structured environments [21,22]. However, Cole [17] and Martin [18] did not explicitly test for power laws nor did they consider alternative models of their movement pattern data. Instead they simply showed that numbers and durations of inactivity intervals appear to be linearly related over about one decade when plotted on log–log scales. This is indicative of power-law scaling but is not compelling. Moreover, the duration of activity bouts were reported to be exponentially rather than power-law distributed. More recently, Bazazi *et al*. [19] employing up-to-date statistical techniques reported on null scale-free intermittent movement patterns in individual desert locust (*Schistocerca gregaria*) nymphs walking within a homogeneous arena. The locust movements were interspersed with pauses, and both the pause and movement bout durations had distributions with power-law tails. The study of Bazazi *et al*. [19] was limited to a single taxon and a single mode of locomotion (walking), and so inferences cannot be drawn about other taxa and forms of locomotion. Wearmouth *et al*. [20] subsequently reported that intrinsic spontaneous intermittency is also evident in the activity patterns of 15 sympatric predator species (including cephalopods, elasmobranch and teleost fishes) under natural and controlled conditions. Waiting time durations in these diverse predatory groups were well approximated by power laws over data ranges up to four orders of magnitude and movement bout durations were exponentially distributed. This study [20] could, however, reflect general responses of marine predators to the presence of external cues, rather than being of the null pattern, as defined here. Scaling exponents are, after all, species-specific, being determined by traits such as foraging mode (active versus ambush predation), body size and prey preference. We therefore tested for the presence of scale-free movements in multiple species under conditions of limited external cues.

## 3. Material and methods

### 3.1 Tethered flight experiments

We analysed the flight patterns in two experimental groups of noctuid moths (Lepidoptera: Noctuidae), and in one experimental group of worker honeybees *A. mellifera* (Hymenoptera: Apidae), tethered to rotational flight mills. The first experimental group involved 255 individuals of 15 species of common British noctuid moths caught from the wild in mercury-vapour light traps on the Rothamsted experimental farm (Hertfordshire, UK). These wild-caught moths were flown on the flight mills at a constant temperature of 18°C, on the night following their capture. Their age is unknown, but only the freshest-looking individuals were selected from traps, so most were assumed to be within a few days of eclosion. The second experimental group of noctuids consisted of second and third generation laboratory-cultured *S. exempta* (African armyworm moths). Insects in this culture were collected from a population outbreak in Zambia in December 2012 and were maintained at Rothamsted under controlled conditions (24°C with a 14 L : 10 D regime). Three hundred individual males and females were flown on the night after emergence (less than 24 h old) at a constant temperature of 24°C. For the third experimental group, 36 actively foraging worker European honeybees (pollen foragers) from colonies maintained at Rothamsted were collected at the hive entrance on the day of the experiment. Having progressed through the in-hive and guarding phases to this last stage of their adult life, these bees can be assumed to be at least three weeks old [23] and were experienced flyers [24,25]. The bees were fed with 10μl of 1 M sucrose solution immediately prior to being flown on the flight mill, thus eliminating energetic constraints that could prevent active flights.

Tethering insects to flight mills to study flight performance and activity patterns has been used in numerous studies over several decades, most notably for studying long-range migratory movements of Lepidoptera (e.g. [26–29]) and honeybee flight performance in the context of physiological and developmental parameters [30,31]. Whether flight mill data realistically reflect flight distances in the wild, where salient external cues can be expected to affect behavioural patterns in various ways, is often debated in these studies. However, as we here focus on the activity patterns occurring under *minimal sensory input*, i.e. behavioural patterns found prior to modulation by external cues, the potential of flight mill experiments to realistically mimic natural flight conditions is of limited relevance here.

In this study, noctuid moths were flown over an 8 h period at night in conditions of complete darkness, on flight mills by tethering them to a horizontal rotating arm that allowed them to fly in a circular trajectory with a circumference of approximately 50 cm [32]. The horizontal arm was attached to a short vertical axle suspended between two magnets so that it had very low frictional drag and rotated freely. An optical sensor with a spatial resolution of 10 cm was used to record the rotation of the arm at 5 s intervals. Customised software was developed to calculate the timing and duration of periods of flight activity and inactivity over the experimental period. The experimental set-up involved 16 identical flight mills run concurrently. Tethered bee flights were recorded in a similar fashion, but over a 6 h period during the day in a laboratory under full-spectrum artificial lighting and a constant ambient temperature of 26°C. Owing to the nature of the bees' mostly visually governed flight, the test-flight mills were enclosed by a screen uniformly striped black and white providing optical flow for flying individuals. Therefore, the bee had significantly more, yet monotone, visual stimulation than the moths which were flown in darkness. Before the experiment bees were kept in groups in small Perspex cages in an incubator at 34°C and provided with 1 M sucrose solution.

Some individual insects (particularly the armyworm moths) undertook sustained uninterrupted flights with relatively few pauses during the experimental period (which may well be indicative of migration flights), while others had activity patterns composed of numerous short flights interrupted by pauses. In this study, we restricted analysis to the 25 wild-caught British moths (from 15 species: 20 males and five females), 19 laboratory-reared armyworm moths (equal sex ratio) and 27 worker honeybees (all females) that had at least 50 pauses during the experimental period.

### 3.2 Experiments of Ueno *et al.* [11]

Ueno *et al*. [11] reported on the temporal organization of rest and activity in 2- to 5-day-old *D. melanogaster*. Walking behaviours of 10 males and 10 females within 5.0 cm diameter arenas placed over food but otherwise featureless were recorded using an infrared camera with an image capture rate of 1 frame s^−1^. The amount of movement was determined by the quantification of the absolute difference between consecutive images. Movement bouts were punctuated by pauses during which movement was not discernible.

### 3.3 Detection of power-law distributions

The Akaike information criterion [33] was used to test whether the distribution of flight and pause time *p*(*t*) in our data provided significant evidence for movement patterns with one of two contrasting patterns:

(1) power laws: *p*_1_(*t*)=*N*_1_*t*^−*μ*^, *b*≥*t*≥*a*

or

(2) exponentials: *p*_2_(*t*)=*N*_2_*e*^−*λt*^*v*^^, *b*≥*t*≥*a*.

where *N*_1_ and *N*_2_ are normalizing factors (which ensure that probabilities add up to 1), *t* is time and where *a*, a constant, marks the start of the tail and where *b* is the duration of the longest record in the dataset. The power-law exponents *μ* and *ν* and the exponential decay rate λ were determined using log maximum-likelihood methods [12]. A simple exponential (associated with Poisson processes) corresponds to *ν*=1 while stretched exponentials (which can resemble power laws) have *ν*<1. The Akaike weight for a power-law distribution can be considered as the weight of evidence in favour of a power-law distribution being the better model of the data, i.e. the Akaike weight for a power law can vary from 0 (no support) to 1 (complete support). The Akaike weights are not, however, indicative of the goodness of fit of the model distributions to the data. Here, the goodness of fit of the best model distribution was quantified by application of the Kolmogorov–Smirnov test. Power-spectra, the square of the magnitude of the Fourier transform of the displacement time-series data, and ‘first-digit’ distributions were also used to test for scale-free Lévy flight characteristics. These analyses can cleanly distinguish between Lévy flight and strong alternative models of movement pattern data [13–15]. The power-law scaling of power-spectra over a range of frequencies *f*, *S*( *f*)∝*f*^−*β*^ with *β*>0 is indicative of scale-free characteristics and suffices to show that movement patterns are not multiphasic (combinations of several different scale-specific processes) that can resemble scale-free motion [12,13]. Scale-specific processes have *β*≈0 and are thus characterized by ‘white’ noise. The first significant digit (i.e. the leading non-zero digit) in the pause durations and flight lengths in a scale-free, Lévy flight must be distributed in a universal way because the units of time and length (be it seconds, hours, inches or metres) must be irrelevant in a movement pattern with no characteristic scales. If the pause durations are 1.2 s, 56.7 s, 0.9 s, 3 .9 s,… then the leading non-zero digits, *n*, are 1, 5, 9, 3,…

## 4. Results

### 4.1 Pause durations and flight lengths of noctuid moths

**Figure 1.**
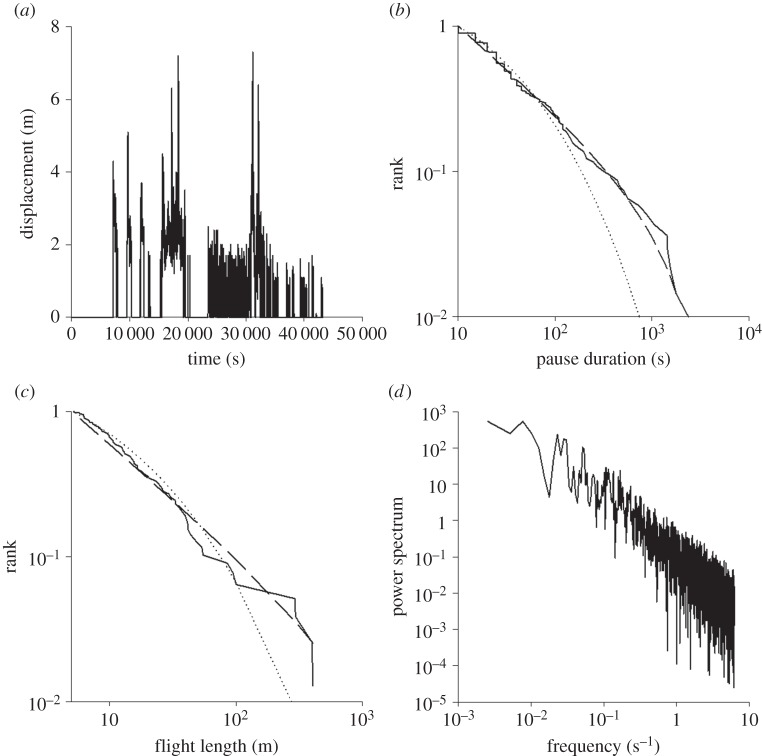
Flight activity in a female noctuid moth, *Apamea monoglypha*. (*a*) An example of a displacement time-series showing intermittency. (*b*) Rank frequency distribution of pause durations longer than 10 s (solid line) together with the best-fit power law (dashed line) and the best-fit stretched exponential (dotted line). The maximum-likelihood estimate for the power-law exponent is *μ*_*P*_=1.57. (*c*) Rank frequency distribution of flight distances longer than 1 m (solid line) together with the best-fit power law (dashed line) and the best-fit exponential (dotted line). The maximum-likelihood estimate for the power-law exponent is *μ*_*F*_=1.57. (*d*) Power-spectrum of the displacement time-series data.

**Figure 2.**
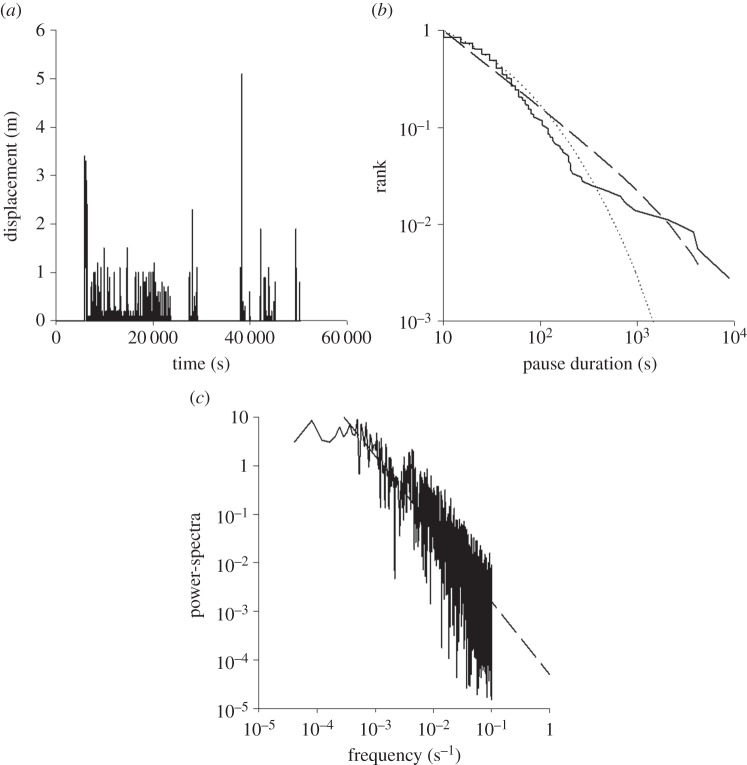
Flight activity in an African armyworm moth (*Spodoptera exempta*). (*a*) An example of a displacement time-series showing intermittency. (*b*) Rank frequency distribution of pause durations longer than 10 s (solid line) together with the best-fit power law (dashed line) and the best-fit stretched exponential (dotted line). The maximum-likelihood estimate for the power-law exponent is *μ*_*P*_=1.7. (*c*) Power-spectrum of the displacement time-series data (solid line) together with the best-fit power (dashed line).

Temporal sequences of flights made by the wild-caught British noctuid moths (15 species) show intermittent patterns (figure 1; electronic supplementary material, figures S1 and S2) with a large proportion of pauses being short, but interspersed with rare very long pauses (approx. 1 h) that are up to 2.5 orders of magnitude greater in duration than the shortest pauses (approx. 5 s). Similarly, short flight distances (approx. 1 m) are interspersed with rare very long flights (approx. 1000 m). This intermittent pattern was evident in all 15 species. The results of our Akaike information criterion and power-spectral tests for all individual moths reveal that intermittent flights have scale-free, Lévy flight characteristics over a range of scales. Pause durations and flight lengths have distributions with power-law scaling and their power-spectra exhibit law scaling time-series typically over more than two orders of magnitude (figure 1; electronic supplementary material, figures S1–S3). In the present instance, the Akaike weights are 1.00 indicating that power laws are convincingly favoured over the alternative exponential (Poisson) distribution as model distributions. In this case, as with the cases presented later, the null hypothesis that the observations come from the best-fit truncated power law cannot be rejected at the 1% level. The maximum-likelihood estimates for the power-law exponents are neither species- nor individual-specific and instead are close to 3/2 (ranging between 1.3 and 1.7). The presence of such power laws is further revealed by power-spectra and first-digit distributions. The power-spectra are also characterized by power-law exponents close to 3/2 (ranging between 1.3 and 1.7; e.g. figure 1*d*). First-digit distributions (see Material and methods) are close to the theoretical expectations for pause durations and flight lengths having distributions with 3/2 power-law scaling. The statistical properties of the flight patterns do not change significantly over time. The best-fit stretched exponentials typically had scaling exponents *ν*<1/2 and so are markedly different from simple exponentials with *ν*=1 associated with Poisson processes. Although not always clearly evident in every individual, 3/2 power-law scaling is manifestly evident when data is pooled for many individuals (electronic supplementary material, figure S2). Intermittent flight patterns with approximate 3/2 power-law scaling characteristics of the kind seen in the wild-caught moths were also evident in analysed flight records of newly eclosed laboratory-reared *S. exempta* that were flown under controlled conditions (figure 2).

### 4.2 Pause durations and flight lengths of honeybees

**Figure 3.**
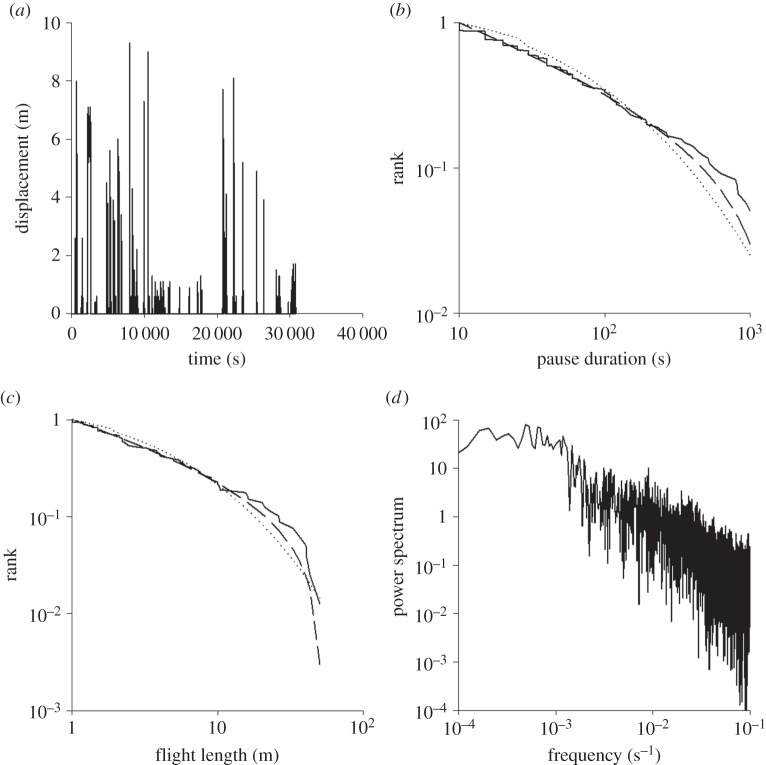
Flight activity in a honeybee, *Apis mellifera*. (*a*) An example of a displacement time-series showing intermittency. (*b*) Rank frequency distribution of pause durations longer than 10 s (solid line) together with the best-fit power law (dashed line) and the best-fit stretched exponential (dotted line). The maximum-likelihood estimate for the power-law exponent is *μ*_*P*_=1.37. (*c*) Rank frequency distribution of flight distances longer than 1 m (solid line) together with the best-fit power law (dashed line) and the best-fit exponential (dotted line). The maximum-likelihood estimate for the power-law exponent is *μ*_*F*_=1.45. (*c*) Power-spectrum of the displacement time-series data.

Of the 36 honeybees recorded on the flight mill, all had intermittent flight patterns and 27 had flight patterns that convincingly favoured power laws over stretched exponential distributions of pause durations (Akaike weight 1.00). One such example is shown in figure 3 and, together with the power-spectra, confirms the presence of scale-free behaviour. Maximum-likelihood estimates for the power-law exponents ranged between 1.0 and 2.5 and had a mean of 1.46. Power-law distributions of flight durations were less prevalent than with the moths, possibly because of a natural variation in the response to the visual stimuli essential to trigger honeybee flights. Although highly monotone and simplified, the presence of these marginal visual cues may affect behavioural patterns in bees with a low response threshold. Thus, the bee flights represent a less rigorous test of the behavioural response to *minimal sensory input* than do nocturnal moths, whose natural flights are mostly guided by olfactory cues but which also fly spontaneously in the complete absence of such cues. Nonetheless, 75% of the bees clearly showed the 3/2 power-law scaling expected for pause durations, with the rest of the bees exhibiting activity patterns modulated by visual cues.

### 4.3 *Drosophila* movement patterns are consistent with the null scale-free template seen in desert locusts, moths and honeybees

Pause durations and walk durations were found to be power-law distributed rather than stretched-exponentially distributed. The maximum-likelihood estimates for the power-law exponents are 1.64 (pauses) and 1.43 (walking). Pause durations are clearly power-law distributed to very good approximation over nearly three decades. The case for movement bout durations being power-law distributed is less convincing but this is perhaps not surprising given the constraining effects of the very small arena.

## 5. Discussion

**Figure 4.**
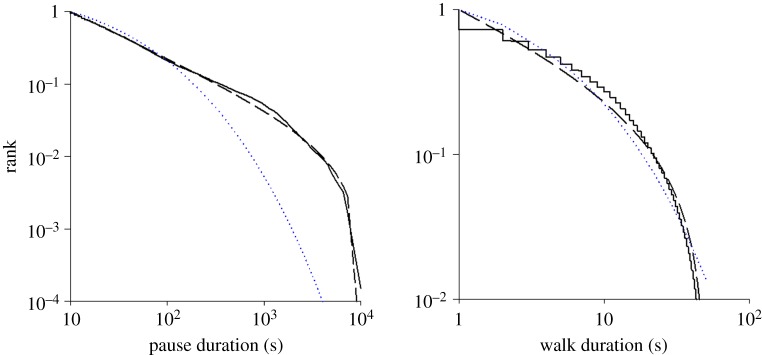
Walking activity patterns of *Drosophila melanogaster* within a 5.0 cm diameter arena. Data for 10 males and 10 females were collected by Ueno *et al.* [11] (solid lines). Rank frequency distributions for pause and walk durations (solid lines) together with the best-fit power laws (dashed lines) and the best-fit stretched exponentials (dotted lines). The maximum-likelihood estimates of the power-law exponents are *μ*_*P*_=1.43 and *μ*_*P*_=1.64.

Animal activity patterns often appear to be haphazard, idiosyncratic and apparently unpredictable. For insects, the impression of randomness can be overwhelming, especially when external stimuli are limited. We present evidence that such movements obey universal statistical laws that are independent of taxon and the mode of locomotion. With the discovery of these laws, it will become possible to disentangle innate, truly random behaviours from the additional influences of the environment and so better understand the complexity of natural movement patterns. Remarkably, the new laws closely mirror the universal statistical laws that characterize daily human activities—laws which are revolutionizing our understanding of human mobility. Understanding the null movement patterns made by organisms under limited external cues is a prerequisite for unravelling the complicated movement patterns that can be observed in natural environments [19]. Identifying the background or foundation condition allows one to assess additional influences of a more complex environment. Here we provided evidence that intermittent scale-free, Lévy flight behaviour in insects is widespread. It was evident in a variety of noctuid moth species (figures 1 and 2; electronic supplementary material, figures S1–S3) and European honeybees, flying under limited external cues (figure 3), and in the walking patterns of *D. melanogaster* in unchanging structured environments with food and water (figure 4). Pause and movement durations were found to have distributions with power-scaling often characterized by scaling close to 3/2. These distributions are quite unlike Poisson distributions, which because of the scarceness of the largest derivations can be characterized just in terms of a mean and a variance. Because power-law distributions cannot be characterized in this simple way, they are indicative of complex underlying processes [12].

The movements of many other animals are interspersed with pauses [34,35]. It has been suggested that pauses provide a variety of energetic benefits and can improve endurance by enabling partial recovery from fatigue [34]. Perceptual benefits could also arise because pauses increase the capacity of the sensory systems to detect relevant stimuli. Several processes, including velocity blur, relative motion detection, foveation, attention and interference between sensory systems could be involved [34]. Searching could, therefore, be saltatory such that ‘scanning’ phases during which resources can be detected alternate with ‘relocation’ phases during which resources cannot be detected, and this has prompted the development of intermittent models of searching and the identification of optimal intermittent searching strategies [36–38]. Nonetheless, none of these potential benefits of pauses account for their durations having distributions with 3/2 power-law tails. However, a 3/2 power-law distribution of waiting times can result from the execution of an intermittent searching strategy in the absence of external cues that promote switching between scanning and relocation phases. For example, suppose that (after an initial cue indicating its presence) a decision about whether or not there is actually a resource in the immediate location is made by accumulating signals/cues (or noise in the absence of a signal because of the intrinsic stochastic nature of the signalling pathway/decision-making circuitry). In addition, suppose that a positive signal (noise) received during a given time window counts as +1 and that the absence of signal (noise) received during a given time window counts as −1, so that a time-series of noise may present itself as 1,1,−1,1,−1,−1 giving the accumulated running totals (evidence) as 1,2,1,2,1,0. When the running total comes to 0, there is no accumulated evidence for the resource being present and a decision is made to move on. For this simple model, it can be shown that decision-making times (pause times) are power-law distributed with exponent 3/2. This is, in fact, a very general result that stems from the Sparre Andersen theorem [39,40] and is not specific to the simple model of decision-making, and is expected even when the noise is temporally correlated [41]. It will typically arise whenever the ‘decision’ to move on is made by accumulating signals (noise) or whenever some internal state-variable that triggers behaviour relaxes noisily towards a threshold and then resets. This may account for the widespread occurrence across taxa of waiting times with distributions having power-law tails with exponents close to 3/2 [12–15,42]. Similarly, noise in the physiological and neurological systems that elicit take-off or landings could result in movement duration (and length) distributions having 3/2 power-law tails. This is not unprecedented as Lévy flight movements in the bacterium *Escherichia coli* in the absence of external stimuli have been attributed to noise in the chemotactic pathway [43] and this is the most parsimonious explanation of the movement patterns. Alternative, but less plausible, explanations for the movement patterns in the absence of external stimuli include: (i) a generalization of Barabási's [1] ‘priority list’ model of bursts and heavy tails in human dynamics; and (ii) optimal Lévy flight searching theory (see the electronic supplementary material for a discussion of these alternatives).

In summary, our new results show that behavioural variability in the form of complex scale-free intermittent movement patterns can be a template for animal motions under limited external cues. We have illustrated that even the simplest of scenarios can result in complex intermittent scale-free movement patterns in a diverse range of taxa. This suggests that the use of Lévy flights as models of animal movement patterns extends beyond random searching movements made in the absence of cues related to target location, and includes random movements more generally. It is conceivable that this template persists, at least in part, in the presence of environmental ‘micro-cues’ that trigger behavioural responses, as exemplified perhaps by the behaviours of T-cells, and rodents in structured environments [21,22,44,45]. This is not unwarranted given that a universal scale-free law underlies daily human activity and that Lévy flights accurately describe human mobility [1,5–7]. The key to understanding these movement patterns evidently lies in identifying the underlying mechanism. Several candidate mechanisms (and potential drivers) were identified, but on balance, the evidence seems to favour the ‘noise in the decision to movement/pause pathway’ mechanism.

Finally, our new result brings about a better understanding of correlated random walks. These stochastic, scale-finite models are the dominant conceptual framework for modelling and interpreting organism movement patterns. Nonetheless, the randomness remains a ‘bugbear’ [46]. Turchin [46, p. 47] wrote ‘Of course, we do not know that animals truly move at random, like flipping coins to decide whether to turn right or left. Each individual could be a perfect automaton, rigidly reacting to environmental cues and its internal states in accordance with some set of behavioural rules. However, even if this were true, we might still choose to model behaviour of such animals stochastically, because we would not have the perfect knowledge of all the deterministic rules driving these animals. The point is that randomness [could be] a modeling convention.’ Our work suggests that scale-free randomness is innate while scale-finite movements are deterministic and result from behavioural responses to external stimuli.

## Supplementary Material

Electronic supplementary material A universal ‘idling-mode’ activity template in flying and pedestrian insects

## Data accessibility

Data has been deposited at Dryad: doi:10.5061/dryad.4b3cd.
