# Supplementary material for: Evidence for a pervasive ‘idling-mode’ activity template in flying and pedestrian insects
Source: R Soc Open Sci. 2015 May 20;2(5):150085. doi: 10.1098/rsos.150085 (PMC4453252; doi:10.1098/rsos.150085)
Supplement: Electronic supplementary material A universal ‘idling-mode’ activity template in flying and pedestrian insects [file rsos150085supp1.doc]

**Electronic supplementary material**

**Evidence for a pervasive ‘idling-mode’ activity template in flying and pedestrian insects**

Andrew M. Reynolds, Hayley B. C. Jones, Jane K. Hill, Aislinn J. Pearson, Kenneth Wilson, Stephan Wolf, Ka S. Lim, Donald R. Reynolds, and Jason W. Chapman

**Text, additional figures and references**

**
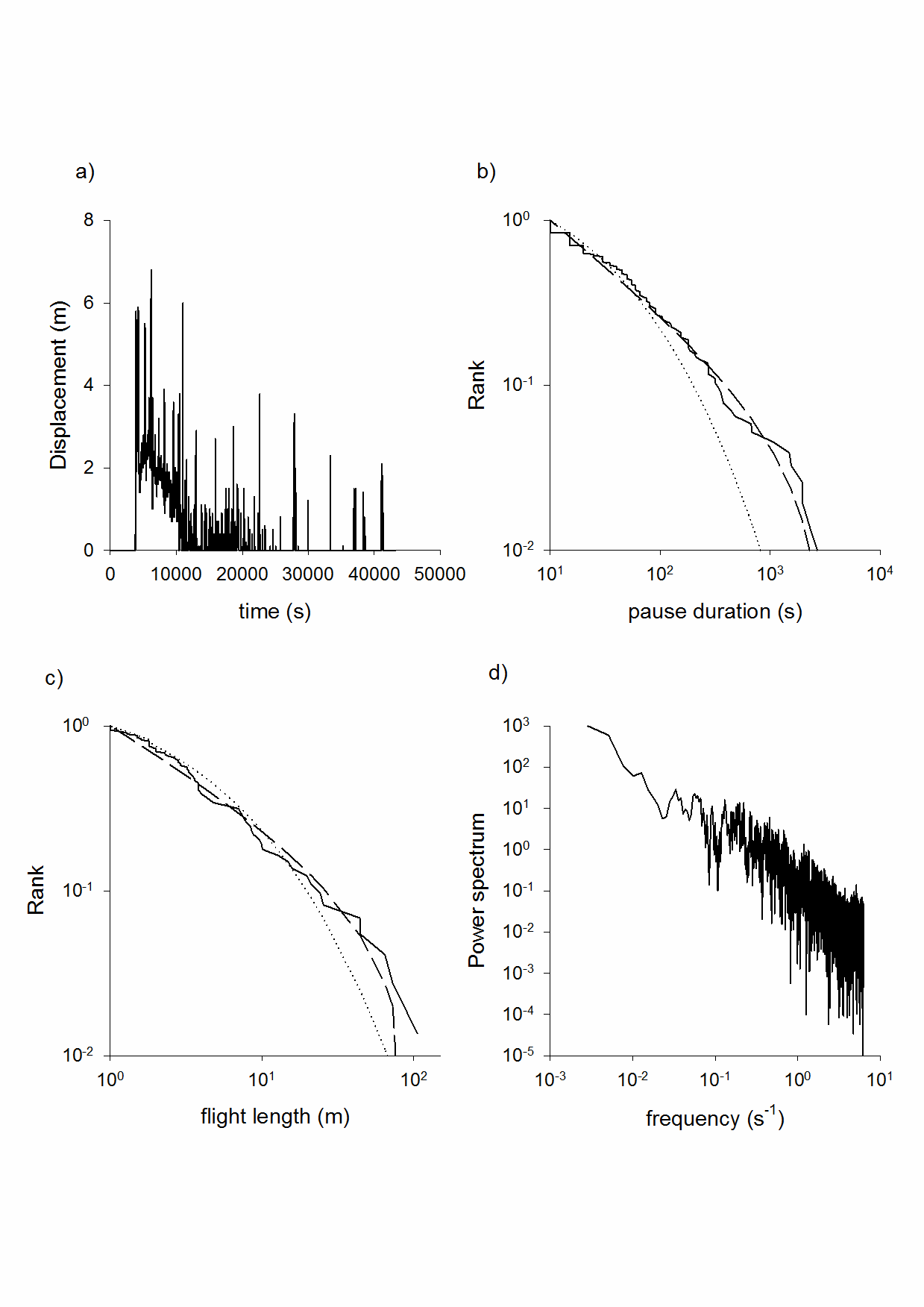
**

**Supplementary Fig. S1.** Flight activity in a female noctuid moth, *Lacanobia oleracea*. (*a*) An example of a displacement time-series showing intermittency. (*b*) Rank frequency distribution of pause durations longer than 10 s (solid-line) together with the best-fit power-law (dashed-line) and the best-fit stretched exponential (dotted-line). The maximum likelihood estimate for the power-law exponent is$\mu_{P}=1.55\pm0.05$. (*c*) Rank frequency distribution of flight lengths longer than 1 m (solid-line) together with the best-fit power-law (dashed-line) and the best-fit exponential (dotted-line). The maximum likelihood estimate for the power-law exponent is$\mu_{F}=1.53\pm0.05$. (*d*) Power-spectrum of the displacement time-series data.


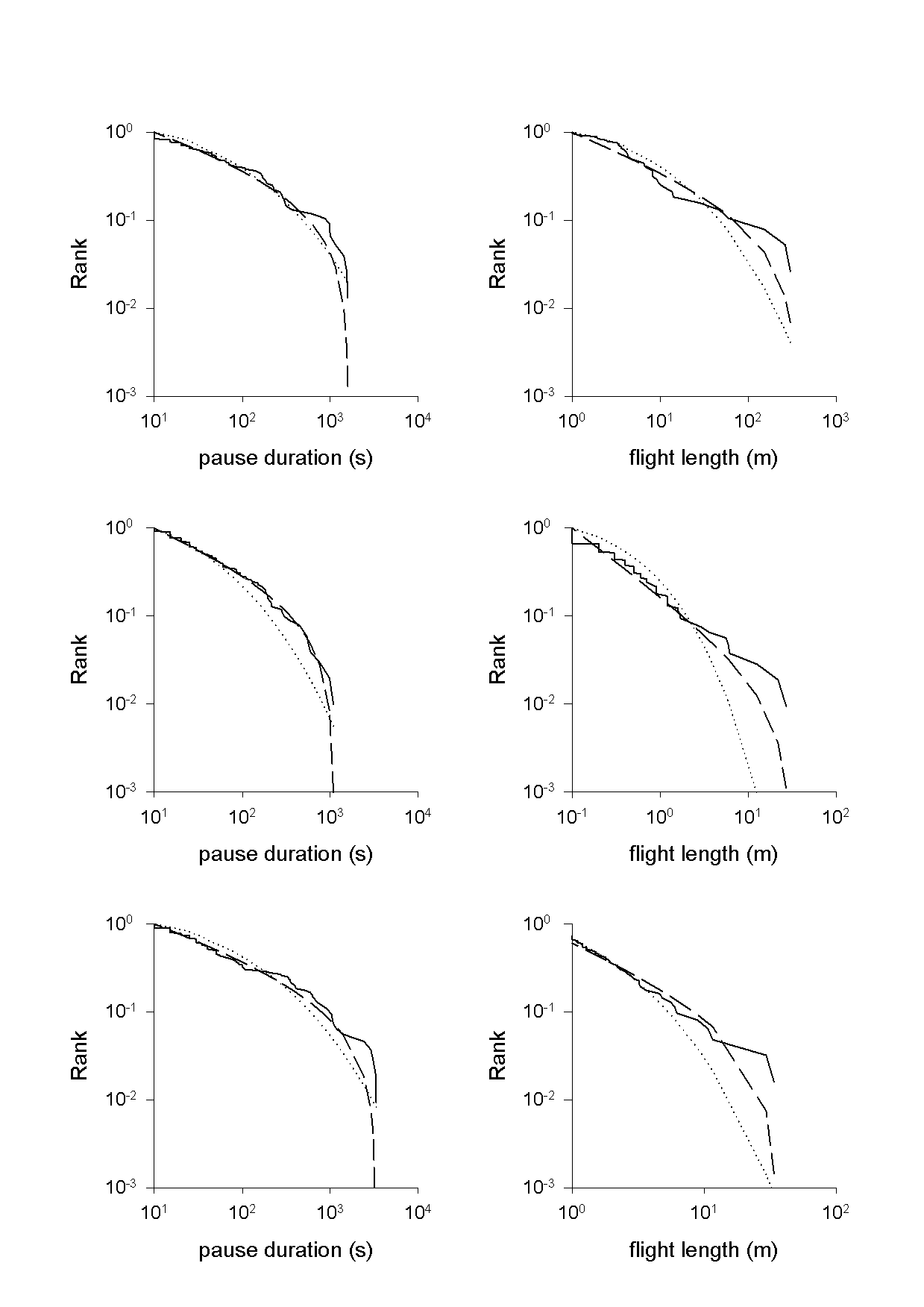


**Supplementary Fig. S2.** Random examples of flight activities of British noctuid moths. Rank frequency distributions of pause durations longer than 10 s (solid-line) and flight lengths together with the best-fit power-law (dashed-line) and the best-fit stretched exponential (dotted-line). In all cases the power-law provides the best fit to the data. The maximum likelihood estimates for the power-law exponents for the pauses are 1.34, 1.41 and 1.74, and for the flight lengths are 1.67, 1.76 and 1.31.


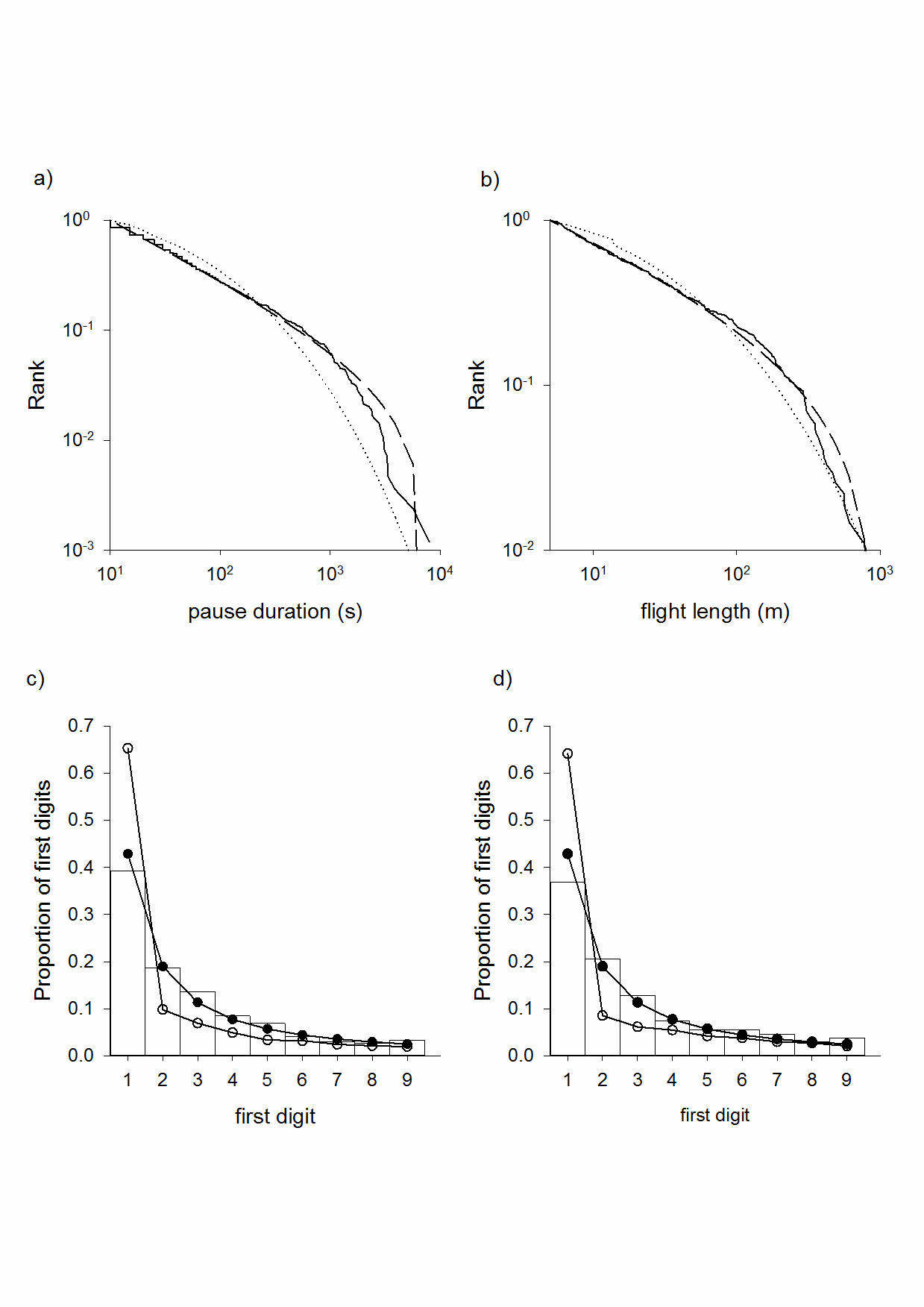


**Supplementary Fig. S3.** Analysis of pooled data for all individual British noctuid moths flown that exhibited intermittent flight patterns. (*a*) Rank frequency distribution of pause durations longer than 10 s (solid-line) together with the best-fit power-law (dashed-line) and the best-fit exponential (dotted-line). The maximum likelihood estimate for the power-law exponent is$\mu_{P}=1.53\pm0.05$. (*b*) Rank frequency distribution of flight distances longer than 1 m (solid-line) together with the best-fit power-law (dashed-line) and the best-fit stretched exponential (dotted-line). The maximum likelihood estimate for the power-law exponent is$\mu_{F}=1.38\pm0.10$. (*c*) First significant digit distribution (histogram) for the pause durations together with the theoretical expectations (●) for the best-fit power-law and the best-fit exponentials (o). (*d*) First significant digit distribution (histogram) for the flight distances together with the theoretical expectations for the best-fit power-laws (●) and the best-fit exponentials (o).

**Alternative candidate explanations for the movement pattern data**

An alternative explanation for the pause distribution can be found in Barabási’s (1) model of bursts and heavy tails in human dynamics. In this model, an agent operates with a priority list of L tasks. Each task on the list has a randomly assigned priority. At each time step the agent selects the highest-priority task from the list and executes it. That task is then removed from the list and a new task is added to the list and given a random priority. In accordance with empirical evidence for email traffic, the model of Barabási (1) predicts that the times that tasks spend on the list are heavy tailed with the power-law exponent close to 1. But a power-law exponent close to 3/2 is attained if the rate at which new tasks are added to the priority list outstrips the rate at which tasks are executed, in accordance with the letter correspondence patterns of Darwin and Einstein (2). The universal law describing human waiting durations also describes the daily behaviors of mice and rats in simple, unchanging arenas with food and water (3–5). It is thus conceivable that the scale-free intermittent motions seen in desert locusts, some noctuid moth species, the European honeybee and *D. melanogaster* results from these animals attempting to resolve a tension between competing and conflicting activities (6, 7). However, this cannot account for the simultaneous occurrence of scale-free pauses and scale-free movement bouts, as the duration of the movement bouts are, in accordance with observations (3–5) predicted to be scale-specific rather scale-free (6). Similarly, the general framework of Proekt *et al*. (5) for understanding how scale invariance may arise in spontaneous animal behaviors provides a candidate explanation for scale-free behaviors in general but cannot account for the ubiquity of a specific scale-free behavior without resorting to fine tuning, as the scaling exponent is free to take on a broad range of values. This shortcoming is also evident in the explanation of the results of Martin *et al*. (8). These authors suggest that the null movement patterns of *Drosophila* reflect neuronal activity of a ‘central pattern generator’ and reported that blocking of synapses established by neurons of the *Drosophila* ellipsoid-body, a substructure of the central complex in the brain, leads to a loss of the fractal properties in the pause structure.

Recent studies have provided seemingly compelling evidence that a diverse range of marine predators, honeybees, bacteria (*E. coli*) and T-cells in mice have Lévy movement patterns and in most of these cases they have been attributed to the execution of innate, evolved optimal searching strategies (9–13), suggesting convergent evolution across taxa. These findings support the Lévy flight foraging hypothesis which posits that “Since [Lévy flights](http://en.wikipedia.org/wiki/Lévy_flight) and walks can optimize search efficiencies, therefore natural selection should have led to adaptations for Lévy flight foraging” (14). The strategy is advantageous when locations of search targets are unknown because proximity cues are either absent or short-ranged (14, 15). Lévy movement patterns in these foragers are characterized by Lévy exponents (power-law exponents) of $\mu\approx2$ (or equivalently by a Hurst parameter H~1 as in the case of little penguins (*Eudyptula minor*) (16)) and may result from interactions with their environments or be underpinned by neuronal activity (17). The null Lévy flight template is different as it is characterized by Lévy exponents of $\mu\approx3/2$; it is thereby discretely different from optimal intermittent searching (17, 18) as $\mu\approx2$ is optimal for both non-destructive and destructive foragers (when searchers have no prior knowledge of the target distribution). Contrary to claims made previously (19), Lévy relocation patterns are not advantageous in intermittent searches when the mean target spacing is known (20). Furthermore, the movements are interspersed by pauses, the durations of which are distributed with 3/2 power-law tails. Such pauses have been identified in the searching patterns of T-cells (13) but seem to be atypical as pauses have not been identified in other foragers with Lévy flight movement patterns. Nonetheless, identifying the mechanisms underpinning the null scale-free movement patterns may help us to understand how optimal Lévy flight behaviors evolved. The origins of these movement patterns are poorly understood and may, unlike the null Lévy template, be dependent upon external cues (17, and references therein).

Despite being sub-optimal in random searches, Lévy flights with $\mu\approx3/2$ could compensate effectively for the occasional occurrence of very long pauses when these pauses are necessary for determining the presence or absence of targets because the decision-making circuitry that triggers movement is noisy. This is because the number of steps (and so the number of pauses) in a Lévy flight search of a given overall length decreases as the Lévy exponent,$\mu$, decreases. As a consequence Lévy flights with $\mu\approx3/2$ (or, more generally with a Lévy exponent $\mu<2$) can represent the best optimal movement pattern given the presence of scale-free pauses, as suggested by the study of Bologna *et al*. (21) and as demonstrated explicitly in the simulation data given in Supplementary Fig. S4 (below). In accordance with the study of Bologna *et al*. (21), total pause time distributions have a 3/2 power-law tail when $1<\mu<3/2$ and a 5/4 power-law tail when $3/2<\mu<3$. The larger, 3/2, power-law tails generates a shorter total pause time and this favors Lévy flight movements with $\mu<3/2$over those with $\mu>3/2$. But this in itself is not sufficient to single out Lévy flight movements with$\mu=3/2$as the optimal strategy, and so account for the observations. Nonetheless, it is possible that neuro-physiological processes can tune up for Lévy flight movements with $\mu=3/2$ (e.g., via the Sparre-Andersen theorem) but not for Lévy flight movements with$\mu<3/2$, making the former ($\mu=3/2$) the biologically accessible movement pattern that best compensates for the pauses. It would be interesting to investigate whether, as suggested by Bologna *et al*. (21), there is a form of resonance between pauses and flights that is beneficial in some random search scenarios, contrary to Aesop’s prejudice that lethargy is always detrimental! A resonance at $\mu=3/2$ coinciding with the observational data for desert locusts, noctuid moth species, European honeybees and *D. melanogaster* is not implausible given the changes in Lévy flight searching-like characteristics that occur with $\mu=3/2$(21, 22). Similarly, the Lévy exponent can be optimized for exploration so that the number of unique landing sites within a given time is maximized and best compensates for pauses but as with searching, the Lévy flights with$\mu=3/2$are not robustly optimal. The situation does not change significantly if it is assumed that free insects do not make unidirectional flights but instead make random scale-finite flights as evidenced, for example, in laboratory studies of bumblebees (*Bombus terrestris*) (23). In this case the 3/2 power-law distribution of flight durations together with the random flights results in $\mu=2$ landing patterns (17). In the absence of pauses, this is an optimal exploration strategy but otherwise it is a sub-optimal strategy (Supplementary Fig. S3).


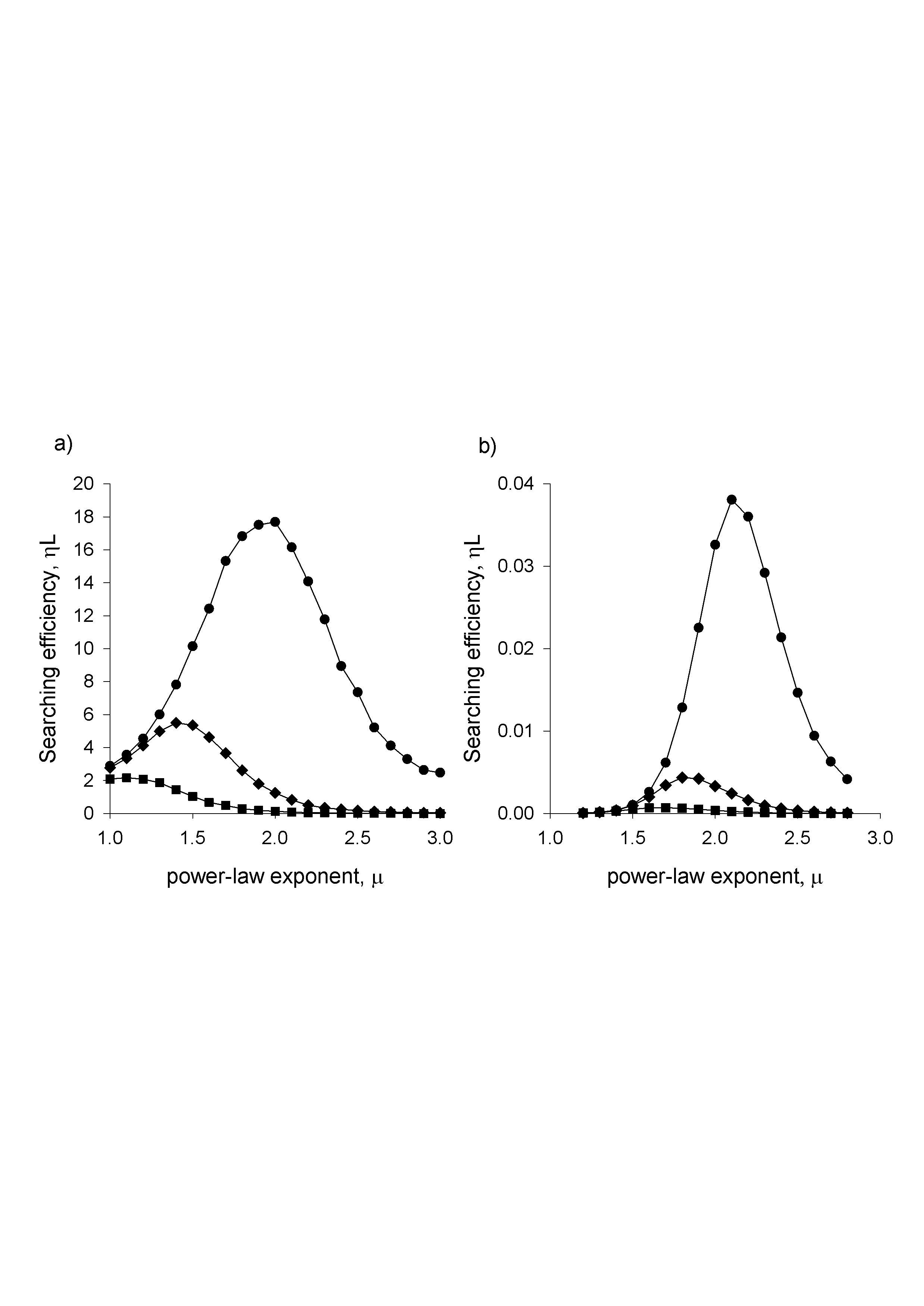


**Supplementary Fig. S4.** Searching efficiencies of Lévy flight movement patterns. Lévy flight patterns with $\mu<2$ can compensate for scale-free pauses (target detection times). The energetic cost of search is taken to be $E=l+gt$where $l$is the total length of the search path and t is the total duration of the pauses in the search. The search efficiency is taken to be$\eta=1/E$. Searching is 1-dimensional and targets are regularly spaced a distance $L=10^{4}$arbitrary space units apart. Targets are detected when they come within the perceptual range of the searcher, a distance of one space unit. Each search begins in the immediate vicinity of a target. Simulation data is shown for $g=0$ (●) (pauses have no energetic cost), 0.1(♦) and 1.0 (■) and for (*a*) continuous searching during movements and for (*b*) searching only during pauses.

**Supplementary References**

1. Barabási A-L (2005) The origin of bursts and heavy tails in human dynamics. *Nature* 435, 207-211.
2. Oliveria JH, Barabási A-L (2005) Darwin and Einstein correspondence patterns. *Nature* 437, 1251-1251.
3. Anteneodo C, Chialvo, DR (2009) Unravelling the fluctuations of animal motion activity. *Chaos* 19(3), 033123.
4. Nakamura, T. *et al*. (2008) Of Mice and Men ‒ universality and breakdown of behavioral organization. *PLoS ONE* 3(4), e2050
5. Proekt A, Banavar JR, Martian A, Pfaff DW (2012) Scale invariance in the dynamics of spontaneous behavior. *Proc Natl Acad Sci USA* 109(26), 10564-10569.
6. Reynolds AM (2011) On the origin of bursts and heavy tails in animal dynamics. *Physica A* 390, 245-249.
7. Sorribes A, Armendariz BG, Lopez-Pigozzi D, Murga C, de Polavieja GG (2011) The origin of behavioural bursts in decision-making circuitry. *PLoS Comput Biol* 7(6), e1002075.
8. Martin J-R, Faure P, Ernst R (2001) The power-law distribution for walking-time intervals correlates with the ellipsoid-body in *Drosophila*. *J Neurogenet* 15, 205-219.
9. Korobkova E, Emonet T, Vilar JMG, Shimizu TS, Cluzel P (2004) From molecular noise to behavioural variability in a single bacterium. *Nature* 428, 574-578.
10. Reynolds AM, et al. (2007a) Displaced honeybees perform optimal scale-free search flights. *Ecology* 88, 1955-1961.
11. Reynolds AM, Smith AD, Reynolds DR, Carreck NL, Osborne JL (2007b) Honeybees perform optimal scale-free searching flights when attempting to locate a food source. *J Exp Biol* 210, 3763-3770.
12. Sims DW, et al. (2008) Scaling laws of marine predator search behaviour. *Nature* 451, 1098-1102.
13. Harris TH, et al. (2012) Generalized Lévy walks and the role of chemokines in migration of effector CD8^+^ T cells. *Nature* 486, 545-548.
14. Viswanathan GM, Raposo EP, da Luz MGE (2008) Lévy flights and superdiffusion in the context of biological encounters and random searches. *Phys Life Rev* 5, 133-150.
15. Viswanathan GM, et al. (1999) Optimizing the success of random searches. *Nature* 401, 911-914.
16. MacIntosh AJJ, Pelletier L, Chiaradia A, Kato A, Ropert-Coudert Y (2013) Temporal fractals in seabird foraging behaviour: diving through the scales of time. *Sci Rep* 3, 1884.
17. Reynolds AM (2013) Beyond optimal searching: Recent developments in the modelling of individual movement patterns as Lévy walks. *Dispersal, individual movement and spatial ecology: A mathematical perspective*, eds Lewis M, Maini P, Petrovskii S (Springer Lecture Notes in Mathematics vol. 2071, Springer-Verlag, Berlin), pp 53-76.
18. Reynolds AM (2006) On the intermittent behaviour of foraging animals. *Europhys Lett* **75(4),** 517-520.
19. Lomholt MA, Tal K, Metzler R, Klafter J (2008) Lévy strategies in intermittent search processes are advantageous. *Proc Natl Acad Sci USA* 105(32), 11055-11059.
20. Bénichou, O., Loverdo, C, Moreau M, Suet PH, Voituriez R (2011) Intermittent search strategies. *Rev Mod Phys* 83, 81-129.
21. Bologna M, Ahat Y, West BJ, Grigolini P (2011) Can intermittent long-range jumps of a random walker compensate for lethargy? *J Phys A* 44, 152003.
22. Berkolaiko G, Havlin, S (1998) Number of distinct sites visited by Lévy flights injected into a d-dimensional lattice. *Phys Rev E* 57, 2549-2552.
23. Lenz F, Chechkin AV, Klages R (2013) Constructing a stochastic model of bumblebee flights from experimental data. *PLoS ONE* 8(3), e59036.
